# Sex‐dependent differences in connectivity patterns are related to episodic memory recall

**DOI:** 10.1002/hbm.26465

**Published:** 2023-08-30

**Authors:** Klara Spalek, David Coynel, Dominique de Quervain, Annette Milnik

**Affiliations:** ^1^ Division of Cognitive Neuroscience Department of Biomedicine University of Basel Basel Switzerland; ^2^ Division of Molecular Neuroscience Department of Biomedicine University of Basel Basel Switzerland; ^3^ Hoekzema Lab, Adult Psychiatry University Medical Centre Amsterdam Amsterdam Netherlands; ^4^ Research Cluster Molecular and Cognitive Neurosciences University of Basel Basel Switzerland; ^5^ Psychiatric University Clinics, University of Basel Basel Switzerland

**Keywords:** diffusion‐weighted imaging (DWI), episodic memory, sex, structural connectome

## Abstract

Previous studies have shown that females typically outperform males on episodic memory tasks. In this study, we investigated if (1) there are differences between males and females in their connectome characteristics, (2) if these connectivity patterns are associated with memory performance, and (3) if these brain connectome characteristics contribute to the differences in episodic memory performance between sexes. In a sample of 655 healthy young subjects (*n* = 391 females; *n* = 264 males), we derived brain network characteristics from diffusion‐weighted imaging (DWI) data using models of crossing fibers within each voxel of the brain and probabilistic tractography (graph strength, shortest path length, global efficiency, and weighted transitivity). Group differences were analysed with linear models and mediation analyses were used to explore how connectivity patterns might relate to sex‐dependent differences in memory performance. Our results show significant sex‐dependent differences in weighted transitivity (*d* = 0.42), with males showing higher values. Further, we observed a negative association between weighted transitivity and memory performance (*r* = −0.12). Finally, these distinct connectome characteristics partially mediated the observed differences in memory performance (effect size of the indirect effect *r* = 0.02). Our findings indicate a higher interconnectedness in females compared to males. Additionally, we demonstrate that the sex‐dependent differences in episodic memory performance can be partially explained by the differences in this connectome measure. These results further underscore the importance of sex‐dependent differences in brain connectivity and their impact on cognitive function.

## Graphical abstract

Women and men differ in their performance on episodic memory tasks. We sought to investigate sex‐dependent differences in connectome characteristics and their relation to memory differences between sexes. Our results show sex‐dependent differences in weighted transitivity, which partially explained the differences in memory between women and men.

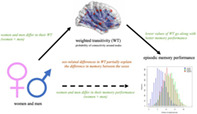

## 1 INTRODUCTION

Sex differences are a highly popular topic, scientifically and socially. An increasing body of research investigates sex differences in a plethora of directions including various aspects of behavior (Geary, 2021; Gur & Gur, 2017; Luoto & Varella, 2021; Proverbio, 2021; Sacher et al., 2013), physiological characteristics (Joye & Evans, 2021; Lombardo et al., 2021; Shepherd et al., 2021), genome‐related differences (Parel & Peña, 2021; Rainville et al., 2021), occurrence of neurological and psychiatric disorders (Anstey et al., 2021; Manosso et al., 2021; Quigley et al., 2021; Salminen et al., 2021; Zhu et al., 2021) as well as in brain function and structure by using different datatypes from various imaging modalities such as functional resonance imaging (fMRI), resting‐state fMRI, volumetric measures, and diffusion‐weighted imaging (DWI; Gur & Gur, 2017; Jahanshad & Thompson, 2017; Sacher et al., 2013).

In a previous publication (Spalek et al., 2015), we specifically observed significant sex‐dependent differences in emotional appraisal and episodic memory performance in a large sample of 3398 healthy young subjects. Specifically, females evaluated particularly negative and positive pictures as emotionally more arousing and outperformed males in the memory recall of all three picture categories (positive, negative, and neutral), with a particular advantage for positive pictures. Two recent reviews (Asperholm et al., 2019; Loprinzi & Frith, 2018) provide further support for a female advantage in various types of episodic memory tasks.

Memory, like most cognitive processes, does not rely on the recruitment of one brain region alone, but instead depends on the successful communication across a network of brain regions (Dickerson & Eichenbaum, 2010; Ritchey & Cooper, 2020; Rugg & Vilberg, 2013). Therefore, the focus on so called cognitive network neuroscience has been receiving increasing attention. In this approach the brain is modeled as a network, or a graph, where each brain region is represented by a node, and the edges of the graph represent interregional connections (Behrens & Sporns, 2012; Bullmore & Sporns, 2009). The strength of these connections is described in weights, which can be defined based on various metrics (Bassett et al., 2011). The edges can represent both functional and structural connections. The structural connectome can be reliably investigated in vivo by using diffusion imaging‐based tractography (Bassett et al., 2011; Owen et al., 2013). Therefore, cognitive network neuroscience aims at understanding the influence of connectome characteristics in a variety of cognitive processes such as emotion, attention, cognitive control, and memory (Medaglia et al., 2015). Specifically, brain connectomics enable to investigate the association of interindividual differences in brain connectivity (Behrens & Sporns, 2012) and relate them to interindividual differences in behavior. The association between episodic memory and structural brain connectivity has so far been investigated mainly in relation with aging (Fjell et al., 2016). Only one study (Coynel et al., 2017) has investigated the association between individual episodic memory performance and the level of structural brain connectivity in healthy young subjects. Additionally, Tunç et al., 2016 were able to distinguish males from females using connectivity as well as behavioral classifiers (derived from performance on a neurocognitive battery including memory tasks). Furthermore, they observed an association between these two classifiers, supporting a link between the two entities.

At the same time, several studies investigate sex‐dependent differences in structural connectomics using DWI data without relating those to behavioral measures (Cahill, 2014; Ingalhalikar et al., 2014; Salehi et al., 2018; Sun et al., 2015; Szalkai et al., 2015). So far, current results point to a more modular function in males and a higher interconnectedness in females. Relating together these findings, we were interested to investigate if differences in brain connectome characteristics of females and males can explain parts of their differences in general episodic memory performance. In a sample of 655 healthy young subjects, we derived brain network characteristics from DWI data using models of crossing fibers within each voxel of the brain and probabilistic tractography. Specifically, we focused on four common measures (Rubinov & Sporns, 2010) on the whole brain level, namely (1) graph strength, representing an estimator of physical wiring cost (sum of edge weights of adjacent edges for each vertex); (2) shortest path length, measuring the length of all the shortest paths from or to the vertices in the network; (3) global efficiency, estimating the ease with which brain regions communicate (average inverse shortest path length); and (4) weighted transitivity, measuring the prevalence of clustered connectivity around individual nodes (probability that adjacent vertices of a vertex are connected). In a first step, we investigated if there were differences between males and females in the network characteristics. Next, we associated the memory performance with these connectome measures. Finally, we run mediation analyses to investigate if differences in connectome measures mediated the association between sex and memory performance.

## 2 MATERIALS AND METHODS

### 2.1 Participants

We analysed data of *N* = 655 subjects, out of which *n* = 391 (60%) were biologically females, with a mean age of 22.62 years (SD = 3.32, range 18–35 years) and *n* = 264 (40%) were biologically males, with a mean age of 23.26 years (SD = 3.46, range 18–35 years). In this study we assessed biological sex. There were no data available on gender identification (i.e., whether biologically recognized males/females identified themselves as men/women). Concerning the use of hormonal contraceptives (HC) and the phase within the menstrual cycle (MC), 199 females were using HC at the time of participating in the study, 155 were not using any type of HC and information was missing from 37 subjects. Detailed information about the specific type of HC was not consistently available. MC‐phase was assessed independently of HC‐use and included the information about the position in the MC (first‐half vs. second‐half). Almost half of the females (*n* = 176) were in the first‐half of their MC, whereas 169 were in the second‐half of their MC. MC data were missing for 39 subjects and 7 subjects reported having no MC. Subjects were recruited from the region of Basel in Switzerland. Sampling strategy was to recruit large samples of healthy young adults, without further restrictions. Advertising was done mainly at the University of Basel and in local newspapers. Subjects were free of any neurological or psychiatric illness, and did not take any medication (except HC) at the time of the experiment. The ethics committee of the Canton Basel approved the experiments. Written informed consent was obtained from all subjects before participation.

The subjects included in this study represent a subset sample of a previously described study (Heck et al., 2014; Spalek et al., 2015), due to DWI data availability (dataset status April 2013). The purpose of the study was to identify biological correlates of cognitive performance by using genetics and imaging techniques in healthy young adults from the general population.

### 2.2 Episodic memory task

We used a picture free recall task to assess episodic memory. For picture encoding, 72 pictures, divided into three valence groups (negative, neutral, and positive), as well as 24 scrambled pictures were presented during the MRI scans by using MR‐compatible LCD goggles (VisualSystem, NordicNeuroLab). On the basis of normative valence scores, pictures from the International Affective Picture System (Lang et al., 1988) were assigned to emotionally negative (2.3 ± 0.6), neutral (5.0 ± 0.3), and positive (7.6 ± 0.4) groups. Eight neutral pictures were selected from an in‐house standardized picture set in order to equate the picture set for visual complexity and content (e.g., human presence). Examples of pictures are as follows: erotica, sports, and appealing animals for the positive valence; bodily injury, snake, and attack scenes for the negative valence; and finally, neutral faces, household objects, and buildings for the neutral condition.

Pictures were presented in an event‐related design, for 2.5 s in a quasi‐randomized order so that a maximum of four pictures of the same category were shown consecutively. A fixation‐cross appeared on the screen for 500 ms before each picture presentation. Trials were separated by a variable inter‐trial period (period between appearance of a picture and the next fixation cross) of 9–12 s (jitter). During the inter‐trial period, subjects rated the presented pictures according to valence (negative–neutral–positive) and arousal (low–middle–high) on a three‐point scale. Four additional pictures showing neutral objects were used to control for primacy and recency effects in memory. The scrambled pictures consisted of a geometrical object in the foreground while the background contained the color information of all pictures used in the experiment (except primacy and recency pictures), overlayed with a crystal and distortion filter (Adobe Photoshop CS3). The object had to be rated regarding its form (vertical‐symmetric‐horizontal) and size (small‐medium‐large).

In an unannounced recall task outside of the scanner, subjects had to freely recall the previously presented pictures, 10 min after the end of picture encoding. An unannounced free recall test was used to avoid recall performance to be influenced by interindividual differences in learning strategies, potentially reflecting non‐mnemonic processes. Participants had to write down a short description (a few words) of the previously seen pictures. Primacy and recency pictures that were remembered as well as training pictures were excluded from the analysis. No time limit was set for this task. Two trained investigators independently rated the descriptions for recall success (inter‐rater reliability >98%). No details were required for correct scoring as pictures were all distinct from each other. The total number of freely recalled pictures was defined as the episodic memory performance phenotype.

### 2.3 DWI analyses

Information about the DWI and T1 acquisition parameters is described in detail in the Data S1 (paragraph “MRI acquisition”).

#### 2.3.1 Preprocessing of diffusion weighted images

Diffusion‐weighted images were preprocessed by using FSL v4.1.7 (Jenkinson et al., 2012; RRID:SCR_002823). Data of 76 participants, for whom slice corruption due to movement was detected (at maximum two directions), were corrected by removing the corrupted directions before further processing (Sharman et al., 2011). Images were first coregistered to the reference unweighted volume (*b* = 0) by using an affine transformation for the correction of head motion and eddy current induced image distortion. Voxelwise model fitting of diffusion orientations was then performed. The local probability distribution of fiber direction was estimated by using *bedpostx*, allowing for automatic estimation of multiple fiber directions within each voxel. At most two directions were estimated (*−n* parameter). The other parameters were left at their default values (ARD weight = 1; burnin period = 1000; number of jumps = 1250; sample every 25; model = 1 [single‐shell]). This approach leads to better sensitivity in the detection of fiber populations as compared to single‐fiber or deterministic approaches (Behrens et al., 2007).

#### 2.3.2 Preprocessing of anatomical T1‐weighted images

T1‐weighted images were segmented into cortical and subcortical structures by using FreeSurfer v4.5 (Fischl et al., 2002) (RRID:SCR_001847). Labeling of the cortical gyri was based on the Desikan‐Killiany atlas (Desikan et al., 2006), yielding 34 cortical regions per hemisphere. Eighty‐two regions (68 cortical and 14 subcortical) were subsequently considered as nodes for the main network analyses. The binary masks defining these nodes were coregistered to the reference unweighted diffusion volume (*b* = 0) using FreeSurfer's *bbregister* command, initialized with the *spm_coreg* command from SPM8 (RRID:SCR_007037). To test for the robustness of our results, we reperformed the analyses using the Destrieux atlas (Destrieux et al., 2010) as an alternative parcellation method. This atlas provides a finer sub‐division of the cortex in 74 sulco‐gyral structures per hemisphere; 162 regions (148 cortical and 14 subcortical) were considered as nodes for this secondary network analysis.

#### 2.3.3 Structural brain network construction: Single subject level, weighted connectivity matrix

**FIGURE 1:**
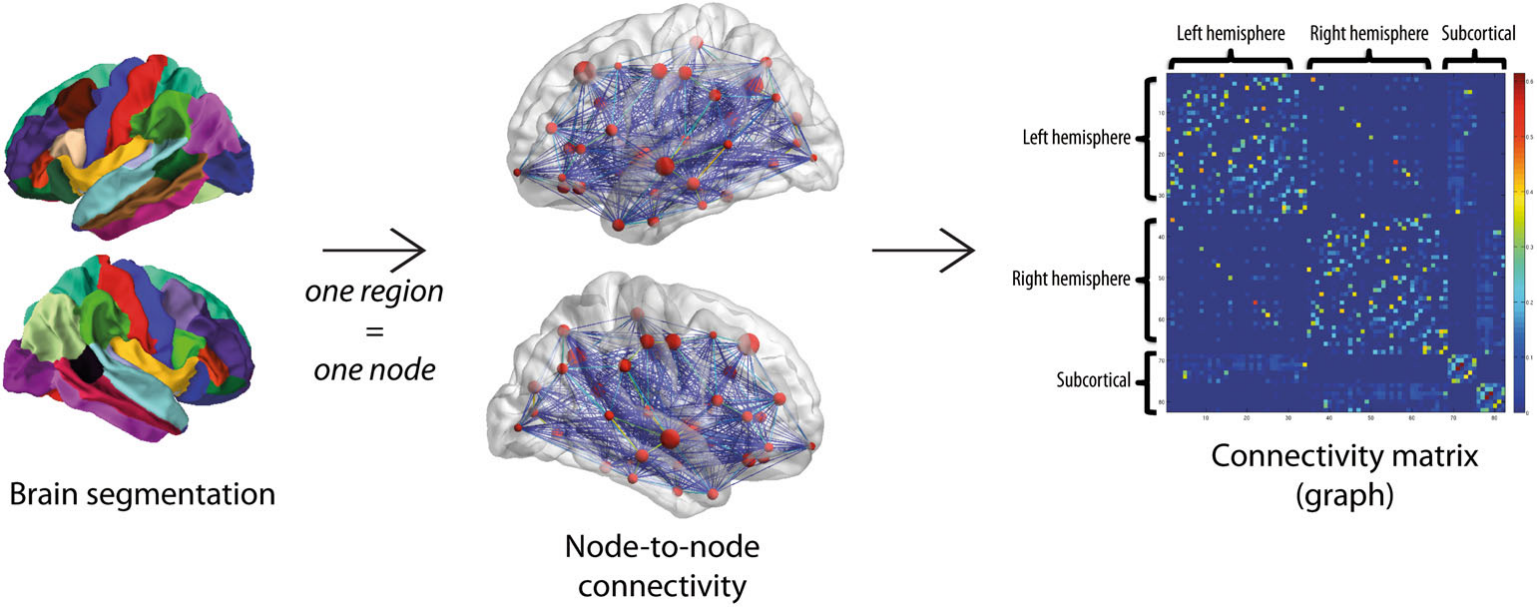
Analysis pipeline. Summary of the preprocessing steps at the single‐subject level. Individual brain segmentation was used as the basis to compute a connectivity matrix from which global, regional, and node‐to‐node network characteristics were computed.

Each anatomical region (as defined above) was considered as one node. Connectivity probability between nodes was estimated by using probabilistic tractography as implemented in *probtrackx2* in FSL v5.0.2 (Behrens et al., 2007). Each node was selected as a seed region, and 5000 sample streamlines were drawn from each voxel within the seed nodes (*−‐nsamples* parameter). Each streamline followed local orientations sampled from the posterior distribution given by *bedpostx*. The streamline stopped when it reached another node (−‐*targetmasks* parameter), or was excluded when it left the brain or passed through the ventricles (−‐*mask* and ‐‐*avoid* parameters, respectively). The node‐to‐node connection probability was represented in a weighted fashion, computed as the number of streamlines successfully reaching another node (−‐*ost* parameter), divided by the total number of drawn streamlines drawn from that seed that were not excluded (waytotal output, Behrens et al., 2007). All other parameters were left to their default values (https://fsl.fmrib.ox.ac.uk/fsl/fslwiki/FDT/UserGuide#PROBTRACKX_‐_probabilistic_tracking_with_crossing_fibres). We focused our subsequent analyses on weighted networks to avoid a potential loss of information when studying binary networks, in which case all non‐null weights would have been set to 1. A whole‐brain symmetrical connectivity matrix was constructed for each subject by averaging the connectivity probabilities obtained from node *i* to *j* and from node *j* to *i* (Gong et al., 2009). In summary, in the main analysis we computed one 82 × 82 weighted connectivity matrix per subject that was used for subsequent analyses (see Figure 1 for an overview).

#### 2.3.4 Structural brain network construction: Population level, discarding spurious connections

A common connectivity threshold at the population level was used to discard spurious connections. A criterion based on the population level enforces consistency in the selection of connections included. More precisely, those connections *C*_*ij*_ for which, across subjects, mean (*C*_*ij*_) + 2 std (*C*_*ij*_) was below a connectivity value of 0.01 were excluded in all subjects. The rationale for using this approach was based on the available suggestions in literature at the time of data preprocessing (2017). Specifically, Gong et al., 2009 used a range of values (0.01–0.1) for their thresholding. We decided to use the lowest threshold of the range as the metrics are computed using weighted connections. Thus, by using the lowest threshold we did not exclude too many connections, and relied on the weighted metrics to put less emphasis on connections that are less strong.

### 2.4 Diffusion‐weighted brain network characteristics

For each subject, the following four brain network characteristics were computed on the whole brain level with the igraph package in R (Csardi & Nepusz, 2006). Namely, (1) graph strength, representing estimator of physical wiring cost (sum of edge weights of adjacent edges for each vertex); (2) shortest path length, measuring the length of all the shortest paths from or to the vertices in the network; (3) global efficiency, estimating the ease with which brain regions communicate (average inverse shortest path length); and (4) weighted transitivity, measuring the prevalence of clustered connectivity around individual nodes (probability that adjacent vertices of a vertex are connected; a variant of the clustering coefficient). G refers to a weighted connectivity matrix, that is, a weighted graph, where *R* = 82 is the number of nodes in the graph and connections between nodes are referred to as *edges*.

### 2.5 Statistical analyses

All calculations were done in RStudio Version 1.3.959 (R Development Core Team, 2011) using the following R packages: linear models–car package (Fox & Weisberg, 2019), mixed models–nlme package (Pinheiro et al., 2017), mediation analyses–MBESS package (Preacher & Hayes, 2008; Preacher & Kelley, 2011), and partial Pearson correlations–ppcor package (Kim, 2015).

We calculated various models in order to investigate the following research questions: (1) Confirmation of the previously reported sex‐dependent memory effect (Spalek et al., 2015) in the herein used subsample. In this model we used the overall memory performance as dependent variable, subject as random effect, sex (female, male; between‐factor), valence category (negative, positive, and neutral; within‐factor), and the interaction term between sex × valence category as contrast of interest (fixed effects). (2) The association of connectome measures with intracranial volume (ICV). We performed for every of the four connectome measures (dependent variables) a separate linear model with sex (female, male; between‐factors) and ICV as independent variable, as well as the interaction term between sex and ICV as contrast of interest. (3) Sex‐dependent differences in the four connectome measures. For every of the four connectome measures (dependent variables) we calculated a separate linear model with sex (female, male; between‐factor) as independent variable. If the overall connectome measure was associated with sex, we additionally extended the analysis to the node‐level. For weighted transitivity node‐level measures are referred to as clustering coefficient. Only *p*‐values which survived Bonferroni multiple comparison correction (correction for number of regions tested) were considered as significant. To test for the robustness of our results, we additionally investigated if other WM‐ or GM‐related variables as well as HC‐use and MC‐phase have an effect on sex‐dependent differences. We therefore recalculated all four models with additional total WM‐ or GM‐volume as covariates, and in the case of HC‐use (HC‐no, HC‐yes, male), and MC‐phase (no‐cycle, first‐half, second‐half, male) as independent variables, respectively. (4) The association of sex‐dependent differences in connectome measures with memory performance. Here, we used the overall memory performance (recalled positive, negative, and neutral pictures together) or the memory performance for positive pictures as dependent variable with subject as random effect, sex (female, male; between‐factor), valence category (negative, positive, and neutral; within‐factor), the connectome measure as independent variable, and all interaction terms as contrasts of interest (fixed effects). For every of the four connectome measures such a model was calculated separately. (5) Mediation of sex effects on memory by differences in connectivity. We calculated a mediation analysis with sex as the independent variable, memory performance as dependent variable and weighted transitivity measure as potential mediator. The ratio of the indirect effect over the direct effect ((*a* × *b*)/*c*') represents the strength of mediation. The percentile confidence intervals for the indirect effects as well as the ratio of the indirect effect over the direct effect are based on a bias‐corrected and accelerated bootstrapping procedure with 10,000 iterations. Significance was assessed by testing whether the confidence intervals of the indirect effects exclude zero in an interval of 95%–99.5%.

All models were estimated by REML (restricted maximum‐likelihood estimation). Age was included as covariate in all models. In models involving the memory performance a batch variable (recall room) was included in addition to age as a covariate. This batch variable refers to a change in the recall condition which occurred throughout the course of the study due to logistic reasons. The first room was very busy and subjects could have been distracted easily or influenced in recalling specific picture contents, while the second room provided a quiet space for performing the task, resulting in a better recall performance. Finally, we corrected for ICV in the models investigating the sex‐dependent differences in the connectome measures by including ICV as covariate. Statistical tests for significance were done with *F* tests. Post‐hoc tests separately for the two sex groups were done with linear models (*t*‐test). In case of group comparisons (males vs. females) we estimated Cohen's *d* as effect size measurement. The estimate of *d* was based on the *t‐*value of the linear models, but not on the mean and standard deviation of the task performance. Therefore, *d* is corrected for the effects of all confounding variables included in the linear model. By convention, *d* = 0.2 is considered to be a small, *d* = 0.5 to be an intermediate and *d* = 0.8 to be a large effect (Cohen, 1992). Due to the factor coding in our main analyses, a positive *d* means that males compared with females had higher values on the dependent variable.

In all partial Pearson correlations age was included as covariate. The batch variable recall‐room was additionally included as covariate when memory effects were tested.

All reported *p‐*values are nominal *p‐*values. To account for the fact that we calculated four main models for the four connectome measures (graph strength, shortest path length, global efficiency, and weighted transitivity), only results with a *p‐*value ≤0.0125 will be called statistically significant.

## 3 RESULTS

### 3.1 Confirmation of valence‐dependent sex effect on memory performance reported by Spalek et al.

Even though we used a substantially reduced sample size compared to Spalek et al., 2015, we still observed overall main effects of valence and sex on memory performance, with positive and negative pictures being remembered better compared to neutral ones, and females outperforming males in the overall memory recall. The significant interaction effect between sex and valence showed that females have a specific advantage for positive pictures (for an overview see Table 1 and Figure S1).

**TABLE 1:** Valence‐dependent sex effect on memory performance in the herein reported data in comparison to one of our previous publications (Spalek et al., 2015).

	Spalek et al., 2015 (2208 females/1370 males)			Current analysis (391 females/264 males)			
Model	*F*‐Value	*p*‐Value		*F*‐Value	*p*‐Value		
Valence category	*F*_(2,6460)_ = 3742.64	1 × 10^−16^		*F*_(2,1308)_ = 846.61	<0.0001		
Sex	*F*_(1,3229)_ = 86.24	1 × 10^−16^		*F*_(1,650)_ = 4.54	0.034		
Sex × valence category	*F*_(2,6460)_ = 35.47	4.4 × 10^−16^		*F*_(2,1306)_ = 7.19	0.0008		

Post‐hoc tests (sex‐dependent)							
	*t*‐Value	*p*‐Value	*d* ^a^	*t*‐Value	*p*‐Value	*d* ^a^	
Recall negative pictures	4.06	5 × 10^−5^	0.15	−0.04	0.9790	−0.003	
Recall neutral pictures	6.16	8.3 × 10^−10^	0.23	1.92	0.0557	0.15	
Recall positive pictures	12.15	1 × 10^−16^	0.45	3.60	0.0003	0.29	

Post‐hoc tests (independent of sex)							
				Difference in means	Lower IC	Upper IC	Adjusted *p*‐value
Neutral vs. negative pictures				−4.21	−4.64	−3.77	3.91 × 10^−11^
Positive vs. negative pictures				0.64	0.20	1.08	0.002
Positive vs. neutral pictures				4.85	4.41	5.29	3.91 × 10^−11^

^a^ Following the factor coding in our previous analyses (Spalek et al., 2015), a positive d means that females had higher memory performance in the respective picture valence category compared with males.

### 3.2 Association of connectome measures with ICV

Combining the two sex groups, all connectome measures were significantly associated with ICV (see Table S1). As expected, sex and ICV were highly correlated (*r* = −0.69, *p* = 3.69 × 10^−93^), with males (mean = 17.64, SD = 1.20) showing larger ICV compared to females (mean = 15.14, SD = 1.36). When testing the association between ICV and connectome measures separately for females and males we see the same directions of effects (for an overview see Table S1 and Figure S2), with graph strength and global efficiency being consistently negatively associated with ICV and shortest path length being consistently positively associated. For weighted transitivity, there is no significant association with ICV when testing separately for females and males. Accordingly, there was no significant interaction between sex and ICV in any of the four connectome measures (*p* ≥ 0.385). To exclude the possibility of confounding effects due to ICV, we corrected for ICV by including total ICV as a covariate in the linear models. For comparison we add the calculations without ICV correction in the Data S1.

### 3.3 Sex differences in connectome measures

**FIGURE 2:**
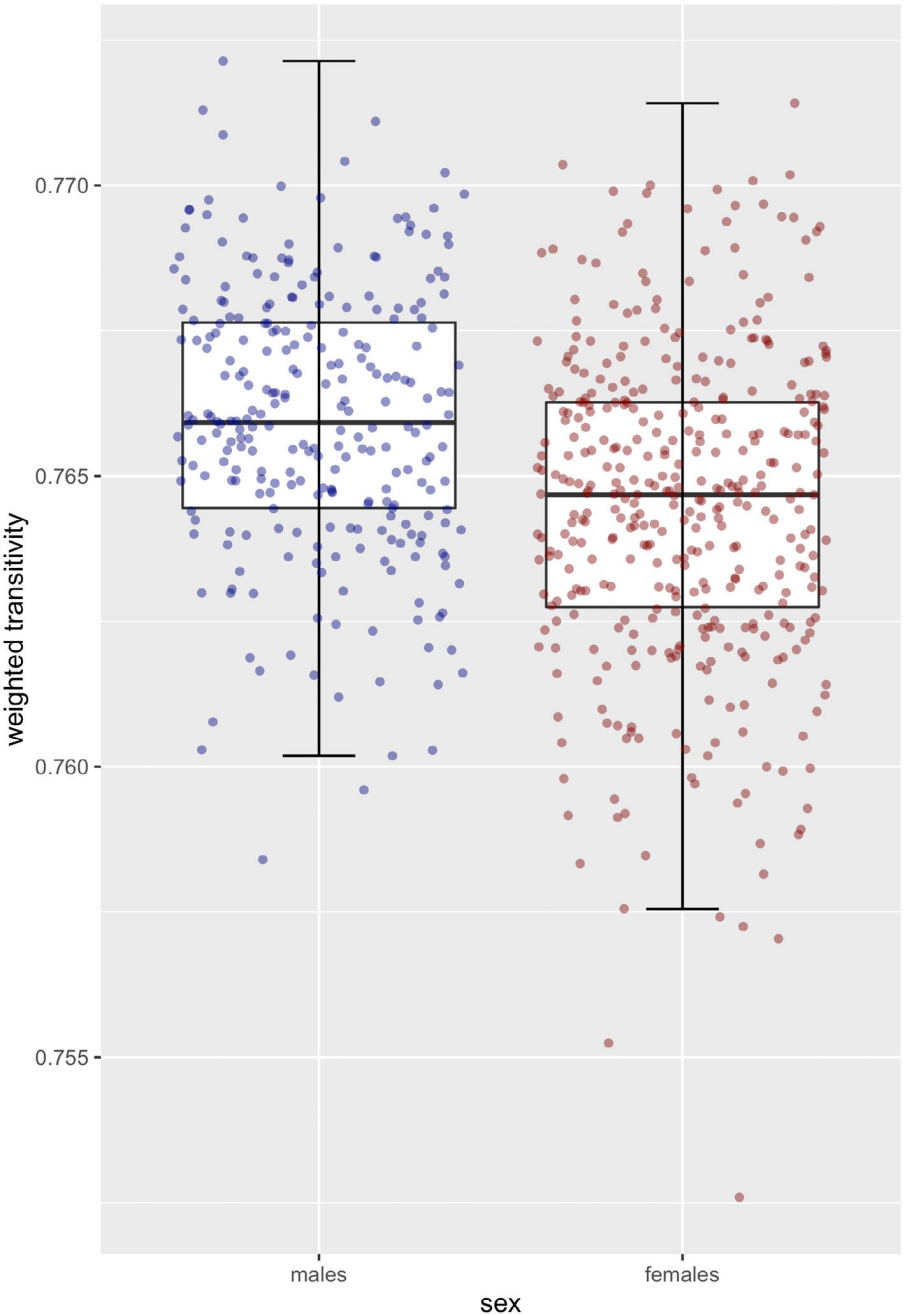
Boxplots with jittered individual subject raw values for weighted transitivity depicted separately per sex group. Red dots = females, blue dots = males.

Next, we investigated if females and males differ in four connectome measures, namely graph strength, shortest path length, global efficiency, and weighted transitivity, even after correction for ICV. Females and males differed significantly in weighted transitivity (*F* = 27.11, *p* = 2.58 × 10^−07^, and *d* = 0.42). Namely, males had higher values in weighted transitivity (mean ± SD, 0.766 ± 0.002) compared to females (0.764 ± 0.003, see Figure 2). For an overview on the results for all measures and the results without ICV correction see Table S2. We observed the same results when using an alternative parcellation (Destrieux atlas, see Table S3). Therefore, we put the focus on weighted transitivity in our subsequent analyses.

To identify regions where the two sexes differ the most, we investigated the sex effect on node‐level for the weighted transitivity (on node‐level referred to as clustering coefficient). Out of the 82 nodes, seven nodes showed a significant sex effect, when correcting for the total number of nodes (*p* < 0.006). These included the following regions: left and right lingual cortex (*F*_L_ = 13.58, *p*_L_ = 0.0002; *F*_R_ = 12.64, *p*_R_ = 0.0004), left postcentral cortex (*F* = 16.39, *p* = 5.77 × 10^−05^), right paracentral cortex (*F* = 12.08, *p* = 0.0005), left and right thalamus (*F*_L_ = 56.79, *p*_L_ = 1.62 × 10^−13^; *F*_R_ = 67.40, *p*_R_ = 1.18 × 10^−15^), and right accumbens (*F* = 16.88, *p* = 4.49 × 10^−05^; for an overview of all results see Table S4).

In order to verify that the sex effect on weighted transitivity was not influenced by other WM‐ or GM‐related variables, we recalculated the model while correcting for the degree (each individual's number of connections, a measure of physical wiring cost) and gray matter volume (GMV). In all models the significant sex effect on weighted transitivity stayed the same (see Table S5). Additionally, we reran the models twice, once with HC‐use (HC‐no, HC‐yes, male) and once with MC‐phase (no‐cycle, first‐half, second‐half, male) so as to investigate if in females the effect further differed between those subgroups. We observed the significant effect between males and females, but no differences within the groups of females neither in HC‐use nor in MC‐phase. For an overview see Table S6.

**FIGURE 3:**
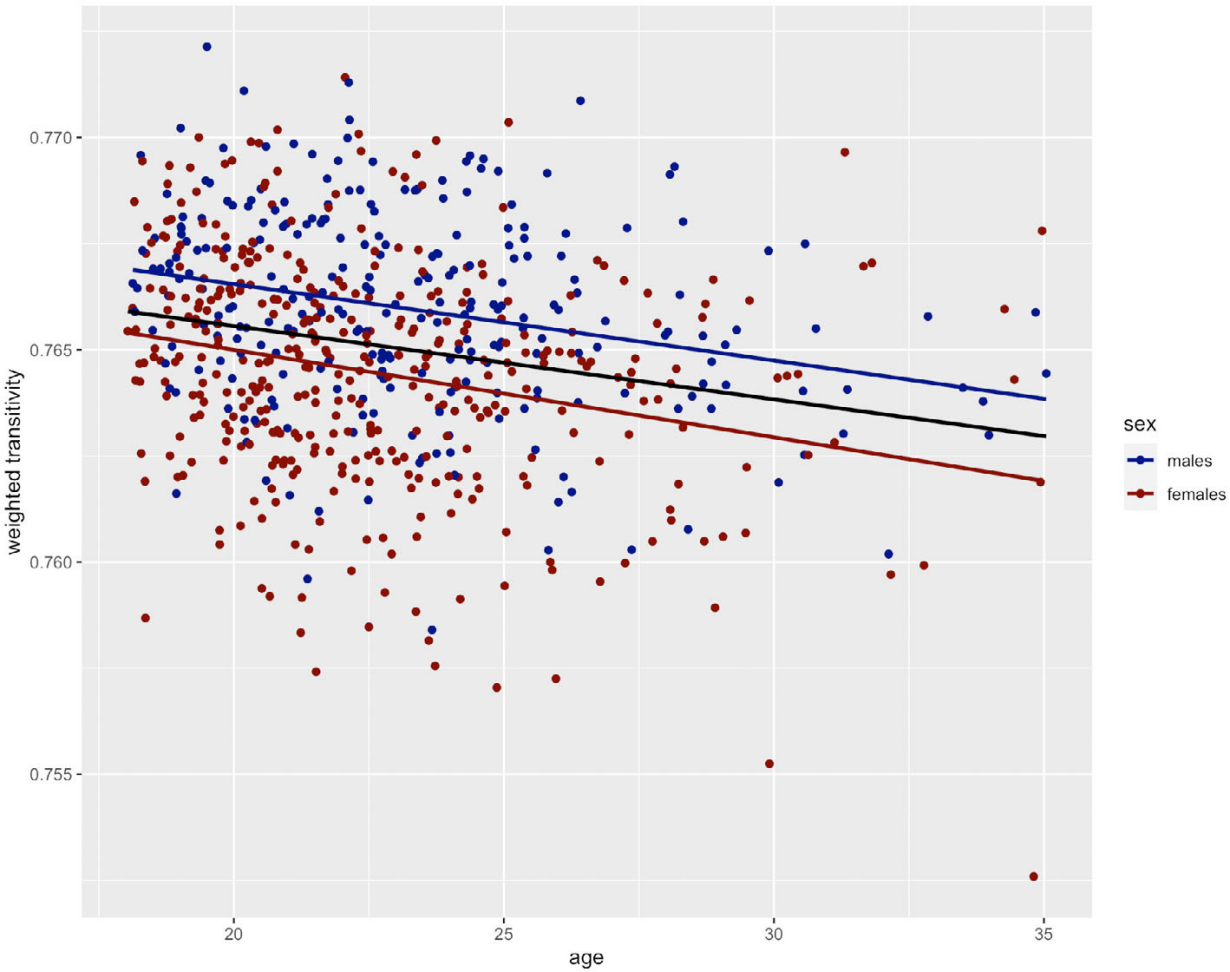
Association between age and weighted transitivity by sex (blue: males, red: females). The regression lines show the association between weighted transitivity and age for all subjects (black line), males (blue line), and females (red line) separately.

Finally, we observed a highly significant association of age with weighted transitivity (*F*_1651_ = 44.74, *p*‐value = 4.86 × 10^−11^), which persisted also when including the additional variables (degree, GMV, HC‐use, MC‐phase). Age was negatively correlated with weighted transitivity (*r* = −0.23, *p*‐value = 2.90 × 10^−09^) and the correlation was significant in both sex groups, when calculated separately (females: *r* = −0.25, *p*‐value = 8.53 × 10^−07^; males: *r* = −0.27, *p*‐value = 9.67 × 10^−06^; see Figure 3).

### 3.4 Association of weighted transitivity with memory performance

Next, we investigated the association between weighted transitivity and overall memory performance, also testing for sex as a potential interacting effect. The three‐way interaction (sex × valence category × weighted transitivity) was not significant (*F* = 0.24, *p* = 0.786). There were no significant two‐way interactions between either sex or valence category and weighted transitivity on memory performance (sex x weighted transitivity: *F* = 0.17, *p* = 0.681; valence category x weighted transitivity: *F* = 1.24, *p* = 0.290). Weighted transitivity had a significant main effect on memory performance (*p* = 0.003). Specifically, weighted transitivity was negatively associated (*r* = −0.12) with memory performance. When analyzing the memory performance of positive pictures only, for which females have compared to males a pronounced memory advantage, results remained the same. Namely, there was no significant two‐way interaction between sex and weighted transitivity (*p* ≥ 0.413). The main effect of weighted transitivity on positive memory performance was significant (*p* = 0.004). Weighted transitivity had a negative association (*r* = −0.12, *p* = 0.002) with memory performance of positive pictures. Without ICV‐correction, we see for all four connectome measures a significant association with memory performance (see Table S7). The results were similar when using an alternative parcellation, with the exception that the main effect of weighted transitivity on memory performance of the positive pictures did not reach significance but was showing a trend only (see Table S8).

### 3.5 Mediation analyses

**FIGURE 4:**
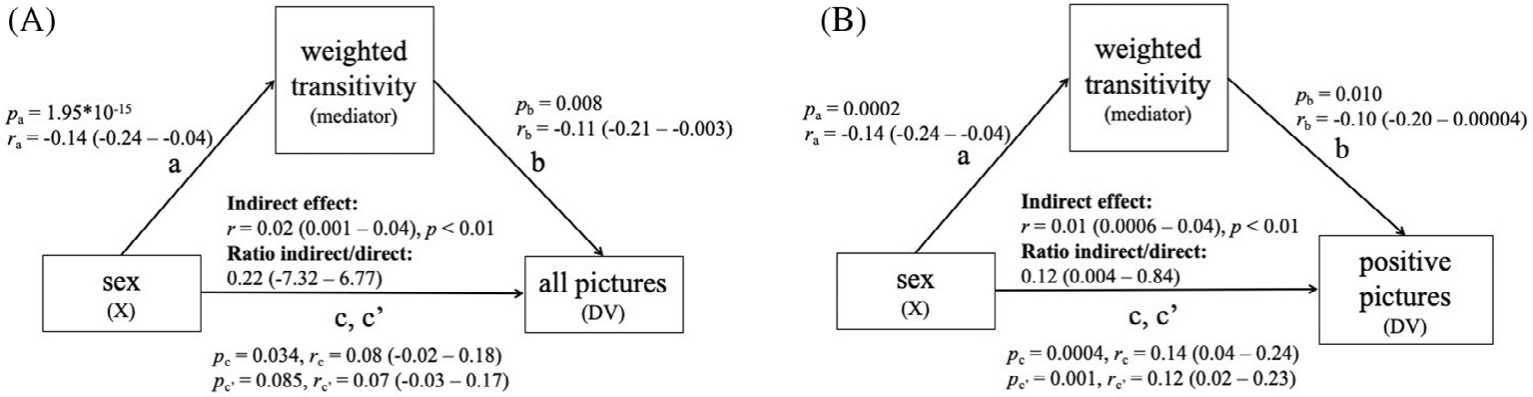
Mediation analysis for the memory performance based on all pictures (A) and based on positive pictures only (B), after correcting for ICV. Path (a) represents the effect of sex on weighted transitivity, whereas path (b) is the effect of the respective connectome measure on memory performance after removing the effect of sex. The indirect effect is computed by multiplying the effects of (a) and (b). Path (c) denotes the effect of the sex on memory performance. Path (c') represents the effect of sex on memory performance while controlling for the indirect effect. Parameters (*r*) show the association strength (±99% confidence interval). Ratio indirect/direct represents the strength of mediation ((a × b)/c'). Parameters (*p*) show significance for path (a), (b), (c), and (c') and indirect effect.

After confirming the established general link between sex and memory, with females outperforming males, a link between sex and weighted transitivity as well as weighted transitivity and memory performance, we were interested if the influence of sex on memory is at least in part mediated by differences in this connectivity measure. Therefore, we calculated a mediation analysis with sex as the independent variable, memory performance as dependent variable and weighted transitivity as potential mediator. In order to control for potentially biasing variables, we removed the effects of age, batch effect and ICV by considering the residuals of a linear model with the respective dependent variable. The association between sex and memory performance for all pictures was partially mediated by weighted transitivity (effect size indirect effect *r* = 0.02; *p* < 0.01, see Figure 4A). The same holds true when focusing on positive pictures only, for which females have a special memory advantage (effect size indirect effect *r* = 0.01; *p* < 0.01, see Figure 4B). Without ICV‐correction, we see for all four connectome measures at least a nominal significant mediation effect on the overall memory performance (Figure S3) and on the memory performance of positive pictures only (Figure S4). When running the mediation analyses with the connectome data based on the Destrieux parcellation, we observe similar results, except for the mediation for positive pictures, which did not reach significance anymore when corrected for ICV (for an overview of the results see Figure S5).

## 4 DISCUSSION

To summarize, our results confirm the previously reported finding about female's advantage in episodic memory tasks compared to males. Additionally, we observed differences in connectome measures between females and males, with females showing lower values in weighted transitivity compared to males. On node‐level, these effects are most pronounced in the left and right lingual cortex, left postcentral cortex, right paracentral cortex, left and right thalamus, and right accumbens. Weighted transitivity is a measure of segregation in the brain. A higher segregation allows more specialized processing within interconnected groups of brain regions (Rubinov & Sporns, 2010). We found that a higher weighted transitivity was negatively associated with memory performance. Differences in weighted transitivity at least partially mediated the association between sex and memory performance. Additionally, weighted transitivity was negatively correlated with age in our cohort of healthy young adults, meaning weighted transitivity decreased with maturity.

The herein observed sex‐dependent differences in weighted transitivity could point to an increased functional segregation in males given that transitivity quantifies the sparsity of the connectome, meaning the ease with which it can be divided into subnetworks (Ingalhalikar et al., 2014). These results are in line with previous research (Cahill, 2014; Ingalhalikar et al., 2014; Szalkai et al., 2015): Female's brains are overall more interconnected suggesting a cross‐module function, whereas males show on average a higher degree of local wiring, appearing to favor a more modular function. On node‐level these effects were mostly pronounced in regions of the reward circuitry (accumbens, thalamus; Bliss & Gardner‐Medwin, 1973; Chen, 2022; Sesack & Grace, 2010), in a region involved in visual encoding (lingual cortex; Machielsen et al., 2000) and in the somatosensory and motor cortices (paracentral and postcentral cortex; Chauhan et al., 2021). Our results show that the effect does not change when correcting for ICV/GMV, and physical wiring cost as well as is independent of the parcellation atlas used, which further underscores and supports its robustness. The difference in weighted transitivity appeared to partly mediate the observed sex‐dependent memory performance differences. Specifically, the fact that females outperform males on the episodic memory recall might be based to some extend on their higher inter‐wiring of brain regions, which allows a more efficient way of communication. It is well‐established that successful memory retrieval requires the conjunct activation of a network of brain regions (Dickerson & Eichenbaum, 2010; Ritchey & Cooper, 2020; Rugg & Vilberg, 2013) further supporting the benefit of a higher interconnectedness and less functional segregation. Thus, these findings complement our previous results (Spalek et al., 2015) as well as existing literature (Coynel et al., 2017; Tunç et al., 2016) by providing a neuronal link for the observed memory advantage of females compared to males. Specifically, we previously identified differences between females and males in emotional episodic memory in a large sample (>3000 subjects). Females outperformed males in overall memory recall (independent of valence of the material), but the difference between them was especially large for the recall of positive pictures. Even though the present sample encompasses a subsample of the sample used in our previous publication, we could still observe these same differences between females and males. While our previous work focused on brain activation, we were able to see a stronger reactivity to negative material, which was at the same time associated with the valence and arousal ratings of the negative pictures. However, we were not able to find a neural correlate explaining the differences in memory recall. The herein identified connectome differences can partially explain the overall memory advantage of females, thus identifying one potential neural correlate. With respect to the pronounced memory advantage of females for positive material, we can just speculate, that this specific valence‐dependent advantage could be related to female hormone effects. We had previously observed difference in memory recall due to HC‐use (Spalek et al., 2019), and there is evidence from the literature that HC‐use and MC‐phase is related to structural and functional brain changes (Dubol et al., 2021; Song et al., 2023). Therefore, we investigated if there were any HC‐use dependent differences associated with the connectome measures. We did not observe any differences related to HC‐use (HC‐users vs. HC‐nonusers) in the whole brain connectome measures. Similarly, MC‐phase (first‐half of cycle vs. second‐half of cycle) did not reveal any differences. These results have to be taken with caution as those variables were not the focus of the study and were thus not assessed in a detailed fashion.

Importantly, we included in our analyses ICV as covariate as it is typically done (Eliot et al., 2021). This approach has the drawback of removing potential sex‐related variance of interest by including a highly colinear covariate in the model. Some more weighted approaches have recently been suggested. Namely, Hyatt et al., 2020 point out the impact of covariate choice including among others sex, ICV and age on analysis results for GMV differences. The authors suggest that the choice of covariates should be guided by the research question, a view also shared by the authors of another review (O'Brien et al., 2006). However, without including ICV, we cannot clearly distinguish between a mere sex‐related effect and a simple mediation by ICV, as a driver of this effect. Therefore, the current golden standard is the use of an ICV‐matched sample. This approach was used already in two publications investigating the sex effect in GMV (Pintzka et al., 2015; Sanchis‐Segura et al., 2020) were the authors concluded that sex differences do exist, though account for a small part of the effects. Nevertheless, we do observe sex‐dependent differences in behavior as well as sex‐dependent differences in connectome measures. This indicates that our analyses without the inclusion of ICV might still capture additional sex‐related differences in connectivity measures.

With respect to the here identified age‐effect on weighted transitivity, it is important to note that global network metrics typically show a U‐shaped trajectory across lifespan (Zhao et al., 2015). For example, the clustering coefficient, which is related to weighted transitivity (Rubinov & Sporns, 2010), shows a decrease in younger age groups but an increase in older ages (Zhao et al., 2015), which might be relevant for the general cognitive decline with increasing age.

Last but not least, although there has been an immense progress in the optimization and methodological improvement of DWI‐based tractography, there are still some limitations to the functional interpretation of results (Assaf et al., 2019; Le Bihan & Iima, 2015). Limitations include an oversimplification of the model for water motion in brain tissue, incomplete knowledge about cause and scale of diffusion limitations, and susceptibility to false negatives (not to identify pathways which exist) as well as false positives (identifying spurious pathways). At the moment, a one‐to‐one relationship between a given diffusion parameter and the underlying tissue structure is not possible.

Our results on sex‐dependent connectome patterns and their association to memory function provide additional knowledge of this relation in healthy subjects. In the light of growing evidence for a change in connectivity patterns in neurological and psychological disorders (for a review see Crossley et al., 2016; Fornito & Bullmore, 2015) and the well‐known observation that females are more vulnerable to develop neuropsychiatric disorders like major depression or anxiety disorders (Holden, 2005), it might be that sex‐dependent differences in brain connectomics contain crucial information for the understanding of pathological patterns involved in these disorders.

## 5 CONCLUSION

The herein observed sex‐dependent differences in brain connectivity provide a neural correlate for the difference between females and males in their episodic memory performance. This extends our previous findings, which highlighted the presence of sex‐dependent differences in emotional appraisal and its neural correlates by identifying related brain activation in mainly motor‐relevant areas (Spalek et al., 2015). Overall, our findings underscore the importance of sex‐dependent differences in brain characteristics and their implications on memory performance.

## CONFLICT OF INTEREST STATEMENT

The authors declare that they have no known competing financial interests or personal relationships that could have appeared to influence the work reported in this article.

## Supporting information


**Data S1.** Supporting Information.

## Data Availability

The data that support the findings of this study are openly available in Sex differences in connectomics at https://osf.io/fe8jn/.
