# Supplementary material for: Sex‐dependent differences in connectivity patterns are related to episodic memory recall
Source: Hum Brain Mapp. 2023 Aug 30;44(17):5612–23. doi: 10.1002/hbm.26465 (PMC10619411; doi:10.1002/hbm.26465)
Supplement: Supplementary file 1 — Data S1. Supporting Information. [file HBM-44-5612-s001.docx]

**SUPPLEMENTAL INFORMATION**

**MRI acquisition**

Measurements were performed on a Siemens Magnetom Verio 3T whole-body MR unit equipped with a twelve-channel head coil. A high-resolution T1-weighted anatomical image was acquired with a magnetization prepared gradient echo sequence (MPRAGE, TR = 2000 ms; TE = 3.37 ms; TI = 1,000 ms; flip angle=8; 176 sagittal slices; FOV = 256 mm; voxel size 1 × 1 × 1 mm3). Diffusion volumes were acquired by using a single-shot echo-planar sequence, and con- sisted of 64 diffusion-weighted volumes (b = 900 s/mm2) and one unweighted volume (b = 0). Acquisition parameters were as follows: TR=9,000ms, TE=82ms, FOV=320mm, GRAPPA R=2.0, voxel size 2.5 × 2.5 × 2.5 mm3.

TABLES AND FIGURES

**Supplementary Table 1.** Association between all four connectome measures and ICV, separately for females and males and for both groups combined.

| **connectome measures** | **females _(_*_n = 391_*_)_** | | **males _(_*_n = 264_*_)_** | | **both groups _(_*_n = 655_*_)_** | |
| --- | --- | --- | --- | --- | --- | --- |
|  | ***r*** | ***p-value*** | ***r*** | ***p-value*** | ***r*** | ***p-value*** |
| graph strength | -0.28 | 3.11x10^-08^* | -0.23 | 0.0002* | -0.40 | 3.01x10^-26^* |
| shortest path length | 0.31 | 5.78x10^-10^* | 0.27 | 1.22x10^-05^* | 0.44 | 6.99x10^-32^* |
| global efficiency | -0.33 | 1.81x10^-11^* | -0.29 | 1.23x10^-06^* | -0.46 | 1.15x10^-35^* |
| weighted transitivity | 0.06 | 0.208 | -0.01 | 0.933 | 0.24 | 6.63x10^-10^* |

** significant associations are marked with an asterisk*

**Supplementary Table 2.** Sex-dependent differences in the four connectome measures in comparison once without ICV-correction and once with ICV-correction.

|  | **MAIN EFFECT SEX** | | | |
| --- | --- | --- | --- | --- |
|  | **connectome measures** | | | |
| **correction ICV** | graph strength | shortest path length | global efficiency | weighted transitivity |
| **no** |  |  |  |  |
| *t*-value | -8.65 | 9.44 | -9.75 | 8.18 |
| *F*-value | 74.74 | 89.09 | 95.05 | 66.87 |
| *p*-value | 4.15 x 10^-17^ | 6.57 x 10^-20^ | 4.68 x 10^-21^ | 1.52 x 10^-15^ |
| *d*^1^ | -0.69 | 0.75 | -0.78 | 0.65 |
| **yes** |  |  |  |  |
| *t*-value | -1.79 | 1.79 | -1.57 | 5.21 |
| *F*-value | 3.20 | 3.21 | 2.45 | 27.11 |
| *p*-value | 0.074 | 0.074 | 0.118 | 2.58 x 10^-07^ |
| *d*^1^ | -0.14 | 0.14 | -0.12 | 0.42 |

*^1^ negative d values reflect higher values in women and positive values reflect higher values in men*

**Supplementary Table 3.** Sex-dependent differences in the four connectome measures in comparison once without ICV-correction and once with ICV-correction based on alternative parcellation with (Destrieux) atlas.

|  | **MAIN EFFECT SEX** | | | |
| --- | --- | --- | --- | --- |
|  | **connectome measures** | | | |
| **correction ICV** | graph strength | shortest path length | global efficiency | weighted transitivity |
| **no** |  |  |  |  |
| *t*-value | -5.47 | 7.12 | -6.42 | 10.93 |
| *F*-value | 29.91 | 50.66 | 41.23 | 119.42 |
| *p*-value | 6.73 x 10^-08^ | 3.22 x 10^-12^ | 2.81 x 10^-10^ | 2.00 x 10^-25^ |
| *d*^1^ | -0.46 | 0.60 | -0.54 | 0.92 |
| **yes** |  |  |  |  |
| *t*-value | -1.59 | 2.28 | -2.06 | 6.34 |
| *F*-value | 2.52 | 5.18 | 4.25 | 40.24 |
| *p*-value | 0.113 | 0.023 | 0.040 | 4.50 x 10^-10^ |
| *d*^1^ | -0.13 | 0.19 | -0.17 | 0.54 |

**Supplementary Table 4.** Sex-dependent differences in 82 nodes of the clustering coefficient.

| **nodes** | **hemisphere (L=left, R=right)** | ***F*-value age** | ***p*-value age** | ***F*-value ICV** | ***p*-value ICV** | ***F*-value sex** | ***p*-value sex** | ***mean*** ± ***SD*** | |
| --- | --- | --- | --- | --- | --- | --- | --- | --- | --- |
|  |  |  |  |  |  |  |  | **females** | **males** |
| accumbens | L | 11.90 | 0.001 | 1.68 | 0.195 | 4.55 | 0.033 | 0.69 ± 0.02 | 0.70 ± 0.02 |
| accumbens | R | 6.24 | 0.013 | 0.09 | 0.768 | 16.88 | 4.49x10^-05^* | 0.71 ± 0.02 | 0.72 ± 0.02 |
| amygdala | L | 1.06 | 0.302 | 3.51 | 0.062 | 1.28 | 0.259 | 0.85 ± 0.02 | 0.85 ± 0.02 |
| amygdala | R | 0.09 | 0.760 | 0.03 | 0.873 | 0.10 | 0.755 | 0.87 ± 0.02 | 0.87 ± 0.02 |
| banks of superior temporal sulcus | L | 2.39 | 0.123 | 0.31 | 0.578 | 0.05 | 0.828 | 0.99 ± 0.01 | 0.99 ± 0.01 |
| banks of superior temporal sulcus | R | 1.60 | 0.206 | 0.07 | 0.787 | 0.89 | 0.347 | 0.91 ± 0.02 | 0.91 ± 0.01 |
| caudal anterior cingulate | L | 47.01 | 0.000 | 0.00 | 0.952 | 6.23 | 0.013 | 0.77 ± 0.02 | 0.77 ± 0.02 |
| caudal anterior cingulate | R | 30.54 | 0.000 | 1.52 | 0.219 | 5.03 | 0.025 | 0.78 ± 0.02 | 0.77 ± 0.02 |
| caudal middle frontal | L | 3.62 | 0.058 | 1.60 | 0.206 | 1.40 | 0.238 | 0.88 ± 0.01 | 0.88 ± 0.01 |
| caudal middle frontal | R | 2.58 | 0.109 | 3.97 | 0.047 | 4.08 | 0.044 | 0.91 ± 0.01 | 0.91 ± 0.01 |
| caudate | L | 2.85 | 0.092 | 0.61 | 0.435 | 2.36 | 0.125 | 0.78 ± 0.02 | 0.78 ± 0.02 |
| caudate | R | 0.01 | 0.919 | 0.00 | 0.950 | 3.58 | 0.059 | 0.78 ± 0.02 | 0.78 ± 0.02 |
| cuneus | L | 0.08 | 0.776 | 0.20 | 0.653 | 0.54 | 0.464 | 0.83 ± 0.01 | 0.83 ± 0.02 |
| cuneus | R | 5.84 | 0.016 | 0.13 | 0.723 | 0.12 | 0.725 | 0.80 ± 0.02 | 0.8 ± 0.02 |
| entorhinal | L | 3.30 | 0.070 | 1.24 | 0.266 | 1.07 | 0.302 | 0.86 ± 0.02 | 0.86 ± 0.02 |
| entorhinal | R | 6.68 | 0.010 | 0.78 | 0.376 | 6.74 | 0.010 | 0.84 ± 0.02 | 0.84 ± 0.02 |
| frontal pole | L | 5.52 | 0.019 | 5.89 | 0.016 | 0.16 | 0.688 | 0.76 ± 0.02 | 0.76 ± 0.02 |
| frontal pole | R | 0.41 | 0.520 | 8.42 | 0.004 | 0.04 | 0.846 | 0.72 ± 0.02 | 0.73 ± 0.02 |
| fusiform | L | 2.64 | 0.104 | 1.37 | 0.243 | 2.16 | 0.142 | 0.87 ± 0.01 | 0.87 ± 0.01 |
| fusiform | R | 5.37 | 0.021 | 9.96 | 0.002 | 0.64 | 0.424 | 0.80 ± 0.01 | 0.80 ± 0.01 |
| hippocampus | L | 9.38 | 0.002 | 24.63 | 0.000 | 0.37 | 0.545 | 0.78 ± 0.01 | 0.78 ± 0.02 |
| hippocampus | R | 5.97 | 0.015 | 7.50 | 0.006 | 0.19 | 0.660 | 0.78 ± 0.01 | 0.79 ± 0.02 |
| inferior parietal | L | 1.64 | 0.201 | 0.34 | 0.560 | 2.71 | 0.100 | 0.76 ± 0.01 | 0.76 ± 0.01 |
| inferior parietal | R | 0.01 | 0.910 | 0.51 | 0.474 | 0.79 | 0.376 | 0.82 ± 0.01 | 0.82 ± 0.01 |
| inferior temporal | L | 0.56 | 0.456 | 0.25 | 0.614 | 0.00 | 0.977 | 0.82 ± 0.02 | 0.82 ± 0.02 |
| inferior temporal | R | 0.26 | 0.612 | 0.06 | 0.810 | 0.39 | 0.533 | 0.82 ± 0.01 | 0.82 ± 0.01 |
| insula | L | 7.54 | 0.006 | 2.29 | 0.131 | 0.00 | 0.969 | 0.79 ± 0.01 | 0.79 ± 0.01 |
| insula | R | 8.15 | 0.004 | 5.14 | 0.024 | 10.27 | 0.001 | 0.74 ± 0.01 | 0.74 ± 0.01 |
| isthmus cingulate | L | 13.14 | 0.000 | 7.11 | 0.008 | 0.00 | 0.999 | 0.72 ± 0.02 | 0.72 ± 0.02 |
| isthmus cingulate | R | 3.74 | 0.054 | 12.69 | 0.000 | 0.07 | 0.793 | 0.72 ± 0.02 | 0.73 ± 0.02 |
| lateral occipital | L | 0.29 | 0.591 | 2.07 | 0.151 | 0.04 | 0.834 | 0.69 ± 0.02 | 0.68 ± 0.02 |
| lateral occipital | R | 0.32 | 0.573 | 4.58 | 0.033 | 0.72 | 0.396 | 0.73 ± 0.02 | 0.73 ± 0.02 |
| lateral orbito-frontal | L | 9.12 | 0.003 | 0.82 | 0.366 | 1.04 | 0.308 | 0.71 ± 0.02 | 0.71 ± 0.02 |
| lateral orbito-frontal | R | 0.81 | 0.367 | 0.78 | 0.377 | 1.67 | 0.197 | 0.74 ± 0.02 | 0.74 ± 0.02 |
| lingual | L | 1.30 | 0.254 | 1.07 | 0.301 | 13.58 | 0.0002* | 0.70 ± 0.02 | 0.71 ± 0.02 |
| lingual | R | 2.72 | 0.100 | 1.12 | 0.290 | 12.64 | 0.0004* | 0.69 ± 0.02 | 0.69 ± 0.02 |
| medial orbito-frontal | L | 2.05 | 0.152 | 1.82 | 0.178 | 4.82 | 0.029 | 0.74 ± 0.02 | 0.74 ± 0.02 |
| medial orbito-frontal | R | 1.05 | 0.305 | 0.52 | 0.472 | 3.77 | 0.053 | 0.73 ± 0.02 | 0.74 ± 0.02 |
| middle temporal | L | 1.33 | 0.249 | 0.73 | 0.393 | 0.43 | 0.513 | 0.79 ± 0.01 | 0.80 ± 0.01 |
| middle temporal | R | 0.66 | 0.418 | 0.01 | 0.941 | 6.68 | 0.010 | 0.78 ± 0.01 | 0.79 ± 0.01 |
| pallidum | L | 0.76 | 0.383 | 0.18 | 0.673 | 5.78 | 0.016 | 0.76 ± 0.02 | 0.77 ± 0.02 |
| pallidum | R | 0.48 | 0.490 | 0.10 | 0.754 | 6.71 | 0.010 | 0.76 ± 0.02 | 0.77 ± 0.01 |
| paracentral | L | 0.25 | 0.619 | 0.72 | 0.395 | 8.85 | 0.003 | 0.85 ± 0.01 | 0.85 ± 0.01 |
| paracentral | R | 1.31 | 0.253 | 0.03 | 0.852 | 12.08 | 0.0005* | 0.78 ± 0.01 | 0.78 ± 0.01 |
| parahippocampal | L | 15.05 | 0.000 | 0.30 | 0.585 | 9.87 | 0.002 | 0.80 ± 0.02 | 0.80 ± 0.02 |
| parahippocampal | R | 10.34 | 0.001 | 0.05 | 0.815 | 2.47 | 0.117 | 0.83 ± 0.02 | 0.83 ± 0.02 |
| pars opercularis | L | 11.87 | 0.001 | 0.03 | 0.855 | 4.86 | 0.028 | 0.92 ± 0.01 | 0.92 ± 0.01 |
| pars opercularis | R | 14.44 | 0.000 | 3.01 | 0.083 | 8.04 | 0.005 | 0.90 ± 0.01 | 0.90 ± 0.01 |
| pars orbitalis | L | 5.82 | 0.016 | 0.85 | 0.358 | 1.85 | 0.174 | 0.90 ± 0.01 | 0.90 ± 0.01 |
| pars orbitalis | R | 0.05 | 0.817 | 1.15 | 0.284 | 3.49 | 0.062 | 0.86 ± 0.02 | 0.86 ± 0.02 |
| pars triangularis | L | 6.40 | 0.012 | 0.32 | 0.573 | 0.05 | 0.824 | 0.87 ± 0.01 | 0.87 ± 0.01 |
| pars triangularis | R | 8.32 | 0.004 | 3.11 | 0.078 | 8.84 | 0.003 | 0.87 ± 0.01 | 0.87 ± 0.01 |
| pericalcarine | L | 1.71 | 0.192 | 1.10 | 0.296 | 3.76 | 0.053 | 0.73 ± 0.02 | 0.73 ± 0.02 |
| pericalcarine | R | 1.57 | 0.211 | 4.11 | 0.043 | 6.18 | 0.013 | 0.78 ± 0.02 | 0.78 ± 0.02 |
| postcentral | L | 0.15 | 0.700 | 2.79 | 0.095 | 16.39 | 5.77x10^-05^* | 0.71 ± 0.02 | 0.72 ± 0.02 |
| postcentral | R | 0.38 | 0.536 | 0.55 | 0.458 | 7.13 | 0.008 | 0.80 ± 0.01 | 0.80 ± 0.01 |
| posterior cingulate | L | 4.58 | 0.033 | 1.49 | 0.222 | 5.96 | 0.015 | 0.77 ± 0.01 | 0.77 ± 0.01 |
| posterior cingulate | R | 10.59 | 0.001 | 3.31 | 0.069 | 1.29 | 0.256 | 0.77 ± 0.01 | 0.77 ± 0.01 |
| precentral | L | 0.49 | 0.483 | 7.83 | 0.005 | 3.51 | 0.061 | 0.70 ± 0.02 | 0.71 ± 0.02 |
| precentral | R | 4.63 | 0.032 | 1.35 | 0.245 | 10.70 | 0.001 | 0.72 ± 0.01 | 0.72 ± 0.01 |
| precuneus | L | 6.11 | 0.014 | 1.16 | 0.282 | 2.19 | 0.140 | 0.55 ± 0.01 | 0.55 ± 0.01 |
| precuneus | R | 3.82 | 0.051 | 1.88 | 0.171 | 7.34 | 0.007 | 0.59 ± 0.01 | 0.60 ± 0.01 |
| putamen | L | 9.47 | 0.002 | 1.11 | 0.292 | 11.76 | 0.001 | 0.69 ± 0.01 | 0.69 ± 0.01 |
| putamen | R | 0.79 | 0.374 | 0.13 | 0.715 | 3.54 | 0.060 | 0.68 ± 0.01 | 0.68 ± 0.01 |
| rostral anterior cingulate | L | 6.37 | 0.012 | 0.02 | 0.875 | 0.02 | 0.877 | 0.78 ± 0.02 | 0.79 ± 0.02 |
| rostral anterior cingulate | R | 0.31 | 0.579 | 0.03 | 0.874 | 0.60 | 0.439 | 0.77 ± 0.02 | 0.78 ± 0.02 |
| rostral middle frontal | L | 0.00 | 0.987 | 5.46 | 0.020 | 0.18 | 0.669 | 0.71 ± 0.02 | 0.71 ± 0.02 |
| rostral middle frontal | R | 1.05 | 0.305 | 0.71 | 0.399 | 0.46 | 0.497 | 0.68 ± 0.02 | 0.68 ± 0.02 |
| superior frontal | L | 5.34 | 0.021 | 1.76 | 0.185 | 1.86 | 0.174 | 0.54 ± 0.01 | 0.54 ± 0.01 |
| superior frontal | R | 2.92 | 0.088 | 1.40 | 0.237 | 1.09 | 0.297 | 0.54 ± 0.01 | 0.54 ± 0.01 |
| superior parietal | L | 5.70 | 0.017 | 0.78 | 0.377 | 0.55 | 0.460 | 0.58 ± 0.02 | 0.58 ± 0.02 |
| superior parietal | R | 0.49 | 0.486 | 1.07 | 0.302 | 8.16 | 0.004 | 0.59 ± 0.02 | 0.60 ± 0.02 |
| superior temporal | L | 1.60 | 0.206 | 0.19 | 0.659 | 0.11 | 0.743 | 0.75 ± 0.01 | 0.75 ± 0.01 |
| superior temporal | R | 1.70 | 0.192 | 2.26 | 0.133 | 9.59 | 0.002 | 0.68 ± 0.01 | 0.69 ± 0.01 |
| supramarginal | L | 3.99 | 0.046 | 0.06 | 0.812 | 1.11 | 0.293 | 0.86 ± 0.01 | 0.86 ± 0.01 |
| supramarginal | R | 8.40 | 0.004 | 0.88 | 0.349 | 1.23 | 0.268 | 0.83 ± 0.01 | 0.83 ± 0.01 |
| temporal pole | L | 4.34 | 0.038 | 0.08 | 0.772 | 1.32 | 0.252 | 0.80 ± 0.02 | 0.80 ± 0.02 |
| temporal pole | R | 9.81 | 0.002 | 4.60 | 0.032 | 1.51 | 0.219 | 0.83 ± 0.02 | 0.83 ± 0.02 |
| thalamus | L | 4.99 | 0.026 | 11.38 | 0.001 | 56.79 | 1.62x10^-13^* | 0.61 ± 0.01 | 0.62 ± 0.01 |
| thalamus | R | 1.10 | 0.295 | 6.75 | 0.010 | 67.40 | 1.18x10^-15^* | 0.62 ± 0.01 | 0.63 ± 0.01 |
| transverse temporal | L | 0.63 | 0.428 | 6.04 | 0.014 | 2.26 | 0.133 | 0.98 ± 0.01 | 0.98 ± 0.01 |
| transverse temporal | R | 1.41 | 0.236 | 1.92 | 0.166 | 0.41 | 0.520 | 0.90 ± 0.01 | 0.90 ± 0.01 |

**Supplementary Table 5.** Sex-dependent differences in weighted transitivity while correcting for degree, GMV.

| **Models** |  | | |
| --- | --- | --- | --- |
|  | variables | *F*-value | *p*-value |
| **Model with ICV and degree** | age | 68.67 | 6.65x10^-16^ |
|  | ICV | 14.15 | 0.0002 |
|  | degree | 381.41 | 3.48x10^-67^ |
|  | sex | 26.73 | 3.11x10^-07^ |
|  |  |  |  |
| **Model with GMV** | age | 26.70 | 3.16x10^-07^ |
|  | GMV | 9.45 | 0.002 |
|  | sex | 22.20 | 3.00x10^-06^ |
|  |  |  |  |
| **Model with GMV and degree** | age | 53.86 | 6.45x10^-13^ |
|  | GMV | 0.02 | 0.877 |
|  | degree | 346.34 | 2.86x10^-62^ |
|  | sex | 8.27 | 0.004 |

**Supplementary Table 6.** Effects of HC-use and MC-phase on sex-dependent differences in weighted transitivity.

| **Main models** | | | | | | | | | | | | | | | | |
| --- | --- | --- | --- | --- | --- | --- | --- | --- | --- | --- | --- | --- | --- | --- | --- | --- |
| **variables** | ***F*-value** | | | ***p*-value** | |  | |  |  | **variables** | ***F*-value** | ***p*-value** | | |  |  |
| age | 1.64 | | | 0.200 | |  | |  |  | age | 0.78 | 0.377 | | |  |  |
| ICV | 7.54 | | | 0.006 | |  | |  |  | ICV | 8.12 | 0.005 | | |  |  |
| HC-use^1^ | 12.18 | | | 6.59 x10^-06^ | |  | |  |  | MC-phase^2^ | 8.62 | 1.33x10^-05^ | | |  |  |
|  |  | | |  | |  | |  |  |  |  |  | | |  |  |
| **Posthocs** | | | | | | | | | | | | | | | | |
| **group comparisons** | | **difference in means** | **lwr CI** | | **upr CI** | | ***p*-value adjusted** | |  | **group comparisons** | **difference in means** | | **lwr**  **CI** | **upr CI** | | ***p*-value adjusted** |
| HC-yes vs. HC-no | | 7.75x10^-06^ | -0.001 | | 0.001 | | 0.999 | |  | MC-1^st^-half vs. MC-nocycle | 0.001 | | -0.002 | 0.003 | | 0.869 |
| men vs. HC-no | | 0.002 | 0.001 | | 0.002 | | 5.04x10^-10^ | |  | MC-2^nd^-half vs. MC-nocycle | 0.001 | | -0.001 | 0.003 | | 0.738 |
| men vs. HC-yes | | 0.002 | 0.001 | | 0.002 | | 5.03x10^-10^ | |  | men vs. MC-nocycle | 0.002 | | 0.000 | 0.005 | | 0.021 |
|  | |  |  | |  | |  | |  | MC-2^nd^-half vs. MC-1^st^-half | 0.000 | | 0.000 | 0.001 | | 0.805 |
|  | |  |  | |  | |  | |  | men vs. MC-1^st^-half | 0.002 | | 0.001 | 0.002 | | 5.06x10^-10^ |
|  | |  |  | |  | |  | |  | men vs. MC-2^nd^-half | 0.002 | | 0.001 | 0.002 | | 5.34x10^-10^ |

*^1^ HC-use subgroups: HC-no (n=155), HC-yes (n=199), men (n=234); MC-phase subgroups: MC-nocycle (n=7), MC-1^st^-half (n=176), MC-2^nd^-half (n=169), men (n=234)*

**Supplementary Table 7.** Association of all four connectome measures with memory performance in comparison once without ICV-correction and once with ICV-correction.

| **connectome measures** | **Main effect of connectome measure on overall picture recall** | | **Main effect of connectome measure on positive picture recall** | |
| --- | --- | --- | --- | --- |
|  | **ICV correction** | **No ICV correction** | **ICV correction** | **No ICV correction** |
| graph strength | *r =* 0.12  *F* = 8.62  *p* = 0.003 | *r =* 0.13  *F* = 10.94  *p* = 0.001 | *r =* 0.12  *F* = 9.66  *p* = 0.002 | *r =* 0.14  *F* = 12.47  *p* = 0.0004 |
| shortest path length | *r =* -0.08  *F* = 4.15  *p* = 0.042 | *r =* -0.10  *F* = 6.14  *p* = 0.013 | *r =* -0.10  *F* = 7.02  *p* = 0.008 | *r =* -0.12  *F* = 9.80  *p* = 0.002 |
| global efficiency | *r =* 0.09  *F* = 5.18  *p* = 0.023 | *r =* 0.11  *F* = 7.49  *p* = 0.006 | *r =* 0.10  *F* = 7.13  *p* = 0.008 | *r =* 0.12  *F* = 10.13  *p* = 0.002 |
| weighted transitivity | *r =* -0.12  *F* = 8.76  *p* = 0.003 | *r =* -0.12  *F* = 9.17  *p* = 0.003 | *r =* -0.11  *F* = 8.37  *p* = 0.004 | *r =* -0.12  *F* = 8.82  *p* = 0.003 |

**Supplementary Table 8.** Association of all four connectome measures with memory performance in comparison once without ICV-correction and once with ICV-correction based on alternative parcellation with (Destrieux) atlas.

| **connectome measures** | **Main effect of connectome measure on overall picture recall** | | **Main effect of connectome measure on positive picture recall** | |
| --- | --- | --- | --- | --- |
|  | **ICV correction** | **No ICV correction** | **ICV correction** | **No ICV correction** |
| graph strength | *r =* 0.16  *F* = 15.25  *p* = 1.05x10^-04^ | *r =* 0.17  *F* = 16.80  *p* = 4.74x10^-05^ | *r =* 0.16  *F* = 15.89  *p* = 0.0001 | *r =* 0.17  *F* = 17.58  *p* = 3.18x10^-05^ |
| shortest path length | *r =* -0.10  *F* = 5.87  *p* = 0.016 | *r =* -0.11  *F* = 7.13  *p* = 0.008 | *r =* -0.10  *F* = 6.60  *p* = 0.010 | *r =* -0.12  *F* = 8.01  *p* = 0.005 |
| global efficiency | *r =* 0.12  *F* = 8.12  *p* = 0.005 | *r =* 0.13  *F* = 9.43  *p* = 0.002 | *r =* 0.12  *F* = 8.97  *p* = 0.003 | *r =* 0.13  *F* = 10.43  *p* = 0.001 |
| weighted transitivity | *r =* -0.10  *F* = 5.88  *p* = 0.016 | *r =* -0.10  *F* = 6.56  *p* = 0.011 | *r =* -0.07  *F* = 3.21  *p* = 0.073 | *r =* -0.08  *F* = 3.77  *p* = 0.053 |

**
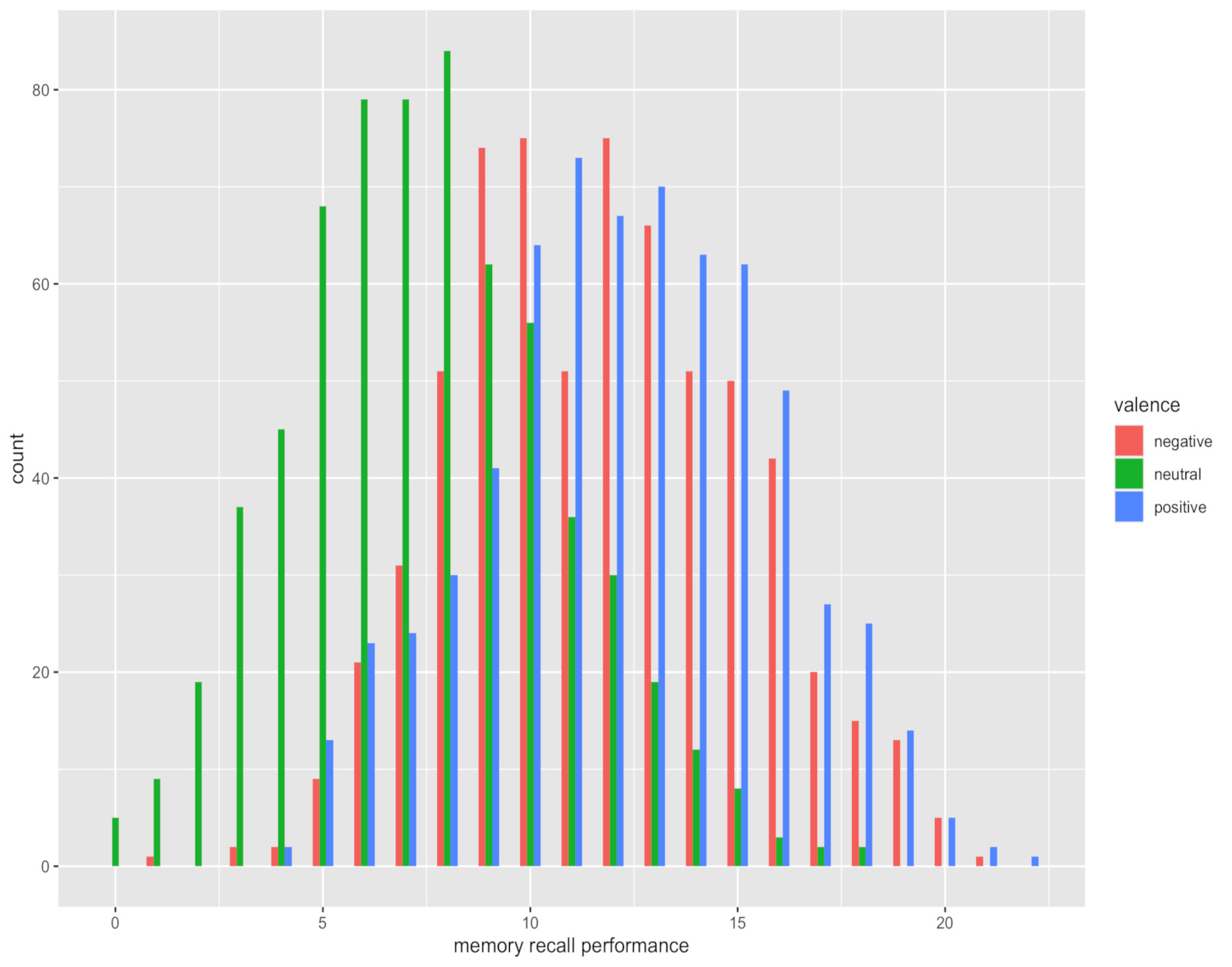
**

**Supplementary Figure 1.** Memory performance distribution across the three valence categories of the presented pictures (negative = red, neutral = green, positive = blue).

*
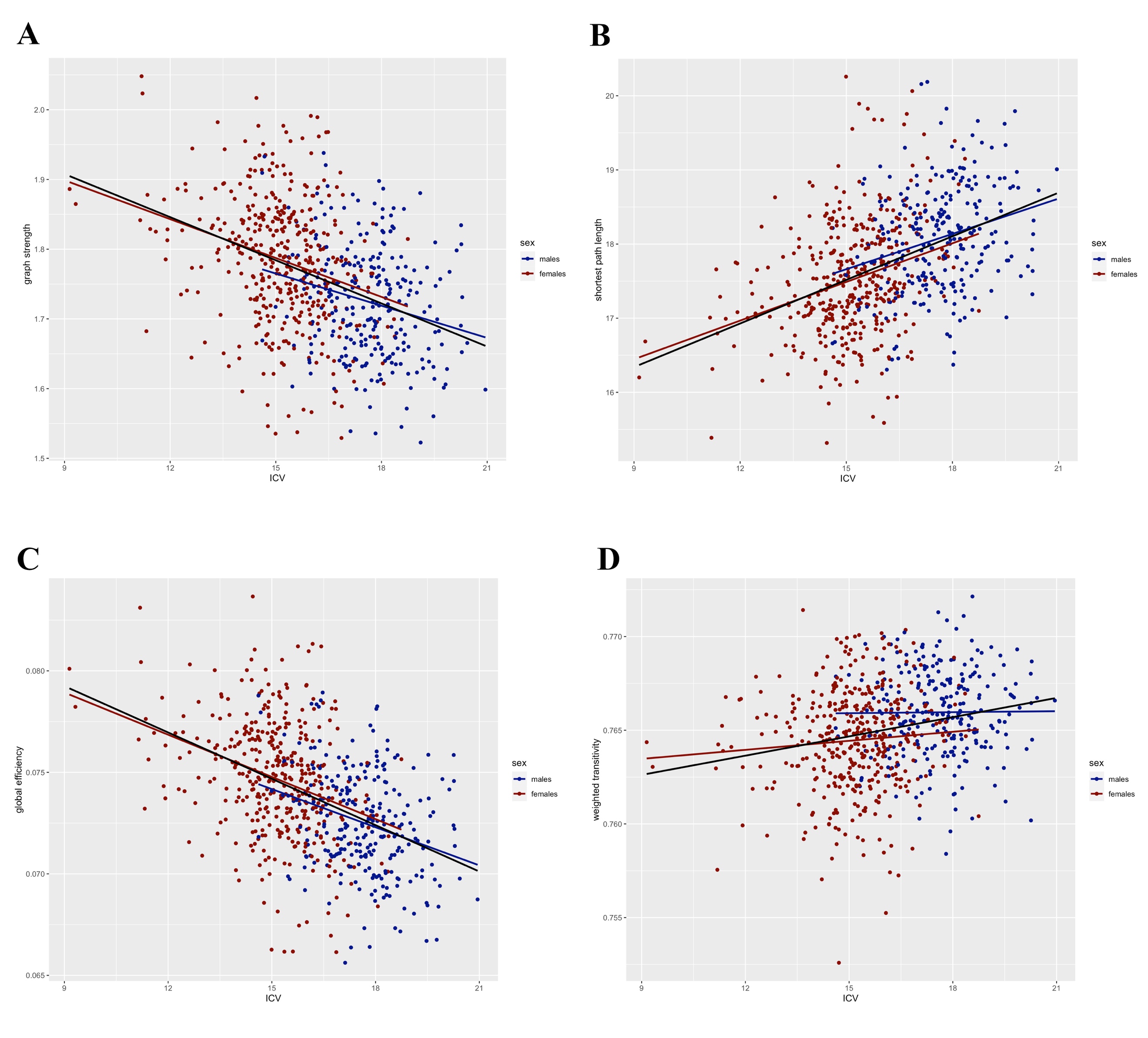
*

**Supplementary Figure 2.** Association of all four connectome measures (A: graph strength, B: shortest path length, C: global efficiency, D: weighted transitivity) with ICV by sex (blue: men, red: women). The regression lines show the association between the respective connectome measure and ICV for all subjects (black line), men (blue line), and women (red line) separately.


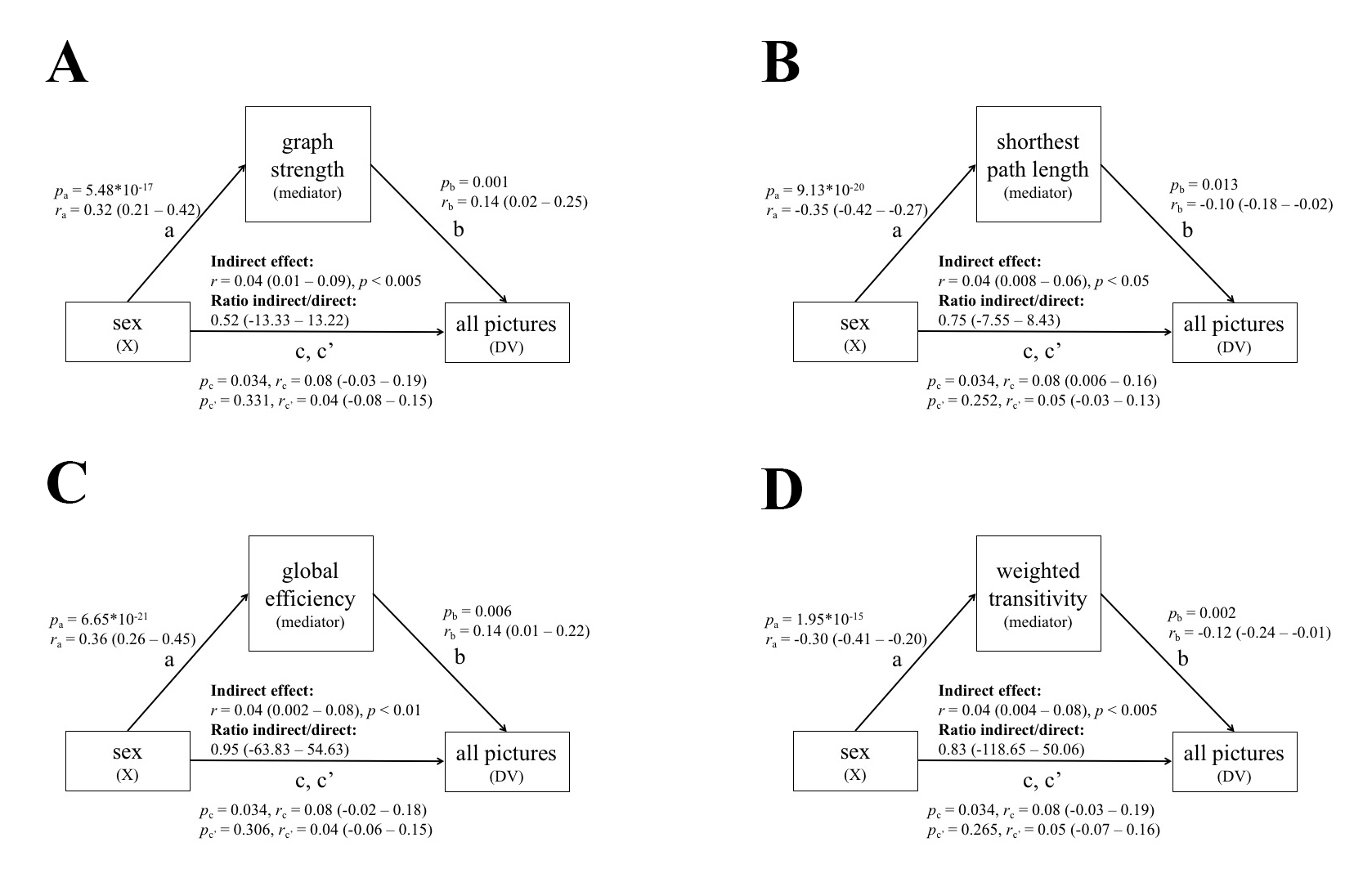


**Supplementary Figure 3.** Mediation analysis for the memory performance based on all pictures without ICV-correction, shown for all four connectome measures (A-D). Path (a) represents the effect of sex on the respective connectome measure, whereas path (b) is the effect of the respective connectome measure on overall memory performance after removing the effect of sex. The indirect effect is computed by multiplying the effects of (a) and (b). Path (c) denotes the effect of the sex on memory performance. Path (c’) represents the effect of sex on memory performance while controlling for the indirect effect. Parameters (r) show the association strength (± 95% - 99.5% confidence interval). Ratio indirect/direct represents the strength of mediation ((a × b)/c’). Parameters (p) show significance for path (a), (b), (c), (c’) and indirect effect.


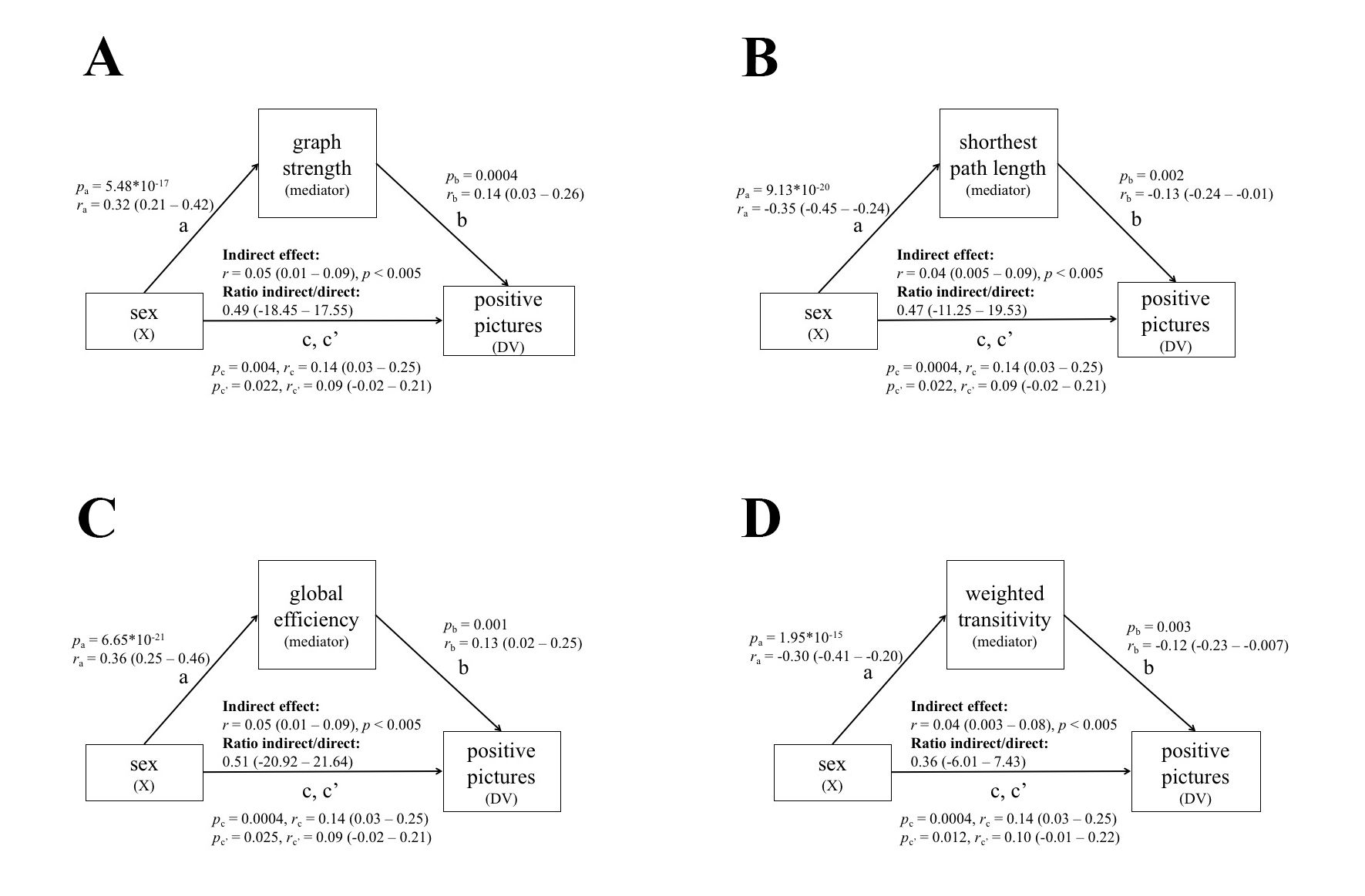


**Supplementary Figure 4.** Mediation analysis for the memory performance based on positive pictures without ICV-correction, shown for all four connectome measures (A-D). Path (a) represents the effect of sex on the respective connectome measure, whereas path (b) is the effect of the respective connectome measure on memory performance of positive pictures after removing the effect of sex. The indirect effect is computed by multiplying the effects of (a) and (b). Path (c) denotes the effect of the sex on memory performance. Path (c’) represents the effect of sex on memory performance while controlling for the indirect effect. Parameters (r) show the association strength (± 99.5% confidence interval). Ratio indirect/direct represents the strength of mediation ((a × b)/c’). Parameters (p) show significance for path (a), (b), (c), (c’) and indirect effect.


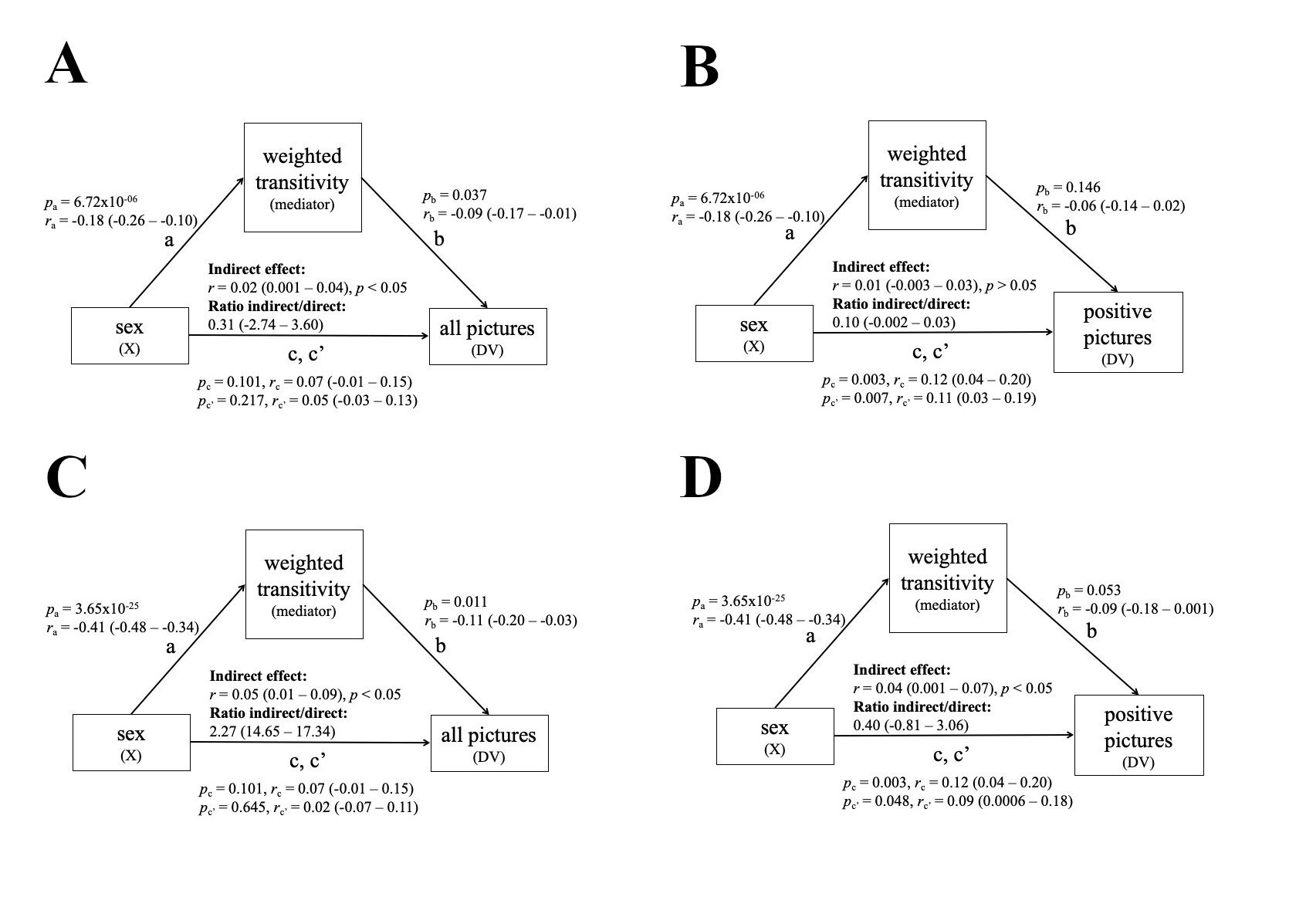


**Supplementary Figure 5.** Mediation analysis for the memory performance based on all pictures (A, C) and based on positive pictures only (B, D) with ICV-correction (A-B) and without ICV correction (C-D) shown for weighted transitivity based on the Destrieux atlas as alternative parcellation method. Path (a) represents the effect of sex on the respective connectome measure, whereas path (b) is the effect of the respective connectome measure on overall memory performance after removing the effect of sex. The indirect effect is computed by multiplying the effects of (a) and (b). Path (c) denotes the effect of the sex on memory performance. Path (c’) represents the effect of sex on memory performance while controlling for the indirect effect. Parameters (r) show the association strength (± 95% - 99.5% confidence interval). Ratio indirect/direct represents the strength of mediation ((a × b)/c’). Parameters (p) show significance for path (a), (b), (c), (c’) and indirect effect.
